# Epigenetic drug library screening identified an LSD1 inhibitor to target UTX-deficient cells for differentiation therapy

**DOI:** 10.1038/s41392-019-0040-2

**Published:** 2019-04-26

**Authors:** Baohong Wu, Xiangyu Pan, Xuelan Chen, Mei Chen, Kaidou Shi, Jing Xu, Jianan Zheng, Ting Niu, Chong Chen, Xiao Shuai, Yu Liu

**Affiliations:** Department of Hematology, State Key Laboratory of Biotherapy and Cancer Center, West China Hospital, Sichuan University, 610041 Chengdu, Sichuan China

**Keywords:** Cancer, Cancer

## Abstract

UTX (also known as KDM6A), a histone 3 lysine 27 demethylase, is among the most frequently mutated epigenetic regulators in myelodysplastic syndrome (MDS) and acute myeloid leukemia (AML). Recent studies have suggested that *UTX* mutations promote MDS and AML by blocking the differentiation of hematopoietic stem and progenitor cells (HSPCs). Here, we performed an epigenetic drug library screening for small molecules able to release the differentiation block on HSPCs induced by *UTX* deficiency. We found that SP2509, a selective inhibitor of LSD1, specifically promoted the differentiation of *Utx*-null HSPCs while sparing wild-type HSPCs. Transcriptome profiling showed that *Utx* loss reduced the expression of differentiation-related and tumor suppressor genes, correlating with their potential roles in HSPC self-renewal and leukemogenesis. In contrast, SP2509 treatment reversed these changes in gene expression in *Utx*-null HSPCs. Accordingly, *Utx* loss decreased H3K4 methylation level probably through the COMPASS-like complex, while LSD1 inhibition by SP2509 partially reversed the reduction of H3K4 methylation in *Utx*-deficient HSPCs. Further, SP2509 promoted the differentiation of *Utx*-null AML cells in vitro and in vivo and, therefore, extended the survival of these leukemic mice. Thus, our study identified a novel strategy to specifically target both premalignant and malignant cells with *Utx* deficiency for differentiation therapy and provided insights into the molecular mechanisms underlying the role of Utx in regulating HSPCs and related diseases.

## Cancer: Restoring differentiation

A small molecule that restores the differentiation of hematopoietic stem and progenitor cells (HSPCs) extends the survival of mice with leukemia. Mutations that impair the activity of UTX, a protein that mediates heritable changes in gene expression by removing methyl groups from histones, prevent the production of blood cells in the bone marrow and have been associated with leukemia. Yu Liu and colleagues at Sichuan University, China, carried out a screen for compounds that target epigenetic regulators and found that SP2509, an LSD1 inhibitor, promoted HSPC differentiation and tumor suppressor gene expression in cells lacking UTX activity. The ability of SP2509 to stop the progression of leukemia in mice suggests that it could be a potential treatment strategy for cancers driven by UTX mutations.

## Introduction

Genes encoding epigenetic modifiers are among the most frequently mutated in hematopoietic malignancies.^1,2^ It has been suggested that dysfunctions in these genes, such as *TET2*, *DNMT3A*, and *IDH1/2*, promote leukemogenesis by blocking the differentiation of hematopoietic stem and progenitor cells (HSPCs).^3–7^ Our previous studies identified Mixed Lineage Leukemia 3 (*MLL3*, officially known as *KMT2C*) on chromosome 7q as a tumor suppressor gene.^8^ Haploinsufficiency of *Mll3* impairs HSPC differentiation and leads to (myelodysplastic syndrome) MDS-like phenotypes. Mll3 (or Mll4), H3K4 mono- and dimethyltransferase, forms the COMPASS-like complex with an H3K27 demethylase Utx (Ubiquitously transcribed tetratricopeptide repeat on chromosome X, officially named as KDM6A) and other components.^9–12^ Therefore, the COMPASS-like complex can regulate target gene expressions by simultaneously modifying both H3K4 and H3K27.^13,14^

Interestingly, *UTX* is highly frequently mutated in human blood malignancies and solid cancers.^15–20^ For example, *UTX* is mutated in approximately 10% chronic myelomonocytic leukemia (CMML) and CMML-derived acute myeloid leukemia (AML) patients and 20% acute lymphoblastic leukemia (ALL) patients.^5,14,21^ *UTX* is essential for multiple biological processes including stem cell self-renewal, embryogenesis, and posterior development.^22–25^ In cancer biology, *UTX* is considered as a tumor suppressor gene of various cancers. *UTX* knockout has been reported to promote pancreatic or lung cancer development.^26,27^ Also, its deficiency could lead to both myeloid and lymphoid malignancies.^28–31^ It is known that *UTX* is required for hematopoiesis. Without it, the frequency and absolute number of both LT-HSCs and ST-HSCs increases due to the impaired cell differentiation.^5,31^

Differentiation therapy has been approved as being effective for hematopoietic malignancies, such as all*-trans*-retinoic acid and arsenic trioxide for acute promyelocytic leukemia.^32–34^ Given the critical role of *UTX* in the regulation of HSPCs, we hypothesized that releasing the differentiation block due to *UTX* deficiencies could benefit patients with *UTX* mutations. To explore potential therapeutic strategies for malignancies with *UTX* deficiencies, we performed an epigenetic drug library screening. We found that SP2509, a putative LSD1 inhibitor,^35^ specifically promoted the differentiation of *UTX* knockout HSPCs while sparing wild-type HSPCs. Mechanistically, UTX, likely through the COMPASS-like complex, facilitates the expression of differentiation-related genes and tumor suppressors associated with increased H3K4 methylation. LSD1 is an H3K4 demethylase.^36^ LSD1 inhibition restores the balance in the H3K4 methylation of target genes in *UTX*-mutant cells and releases them from differentiation block. Intriguingly, SP2509 had similar effects on *UTX*-null AML cells and extended the lifespan of animals with *UTX*-deficient-driven leukemia.

## Results

### Epigenetic drug library screening identified candidate small molecules for specifically promoting the differentiation of *Utx*-null HSPCs

**Fig. 1:**
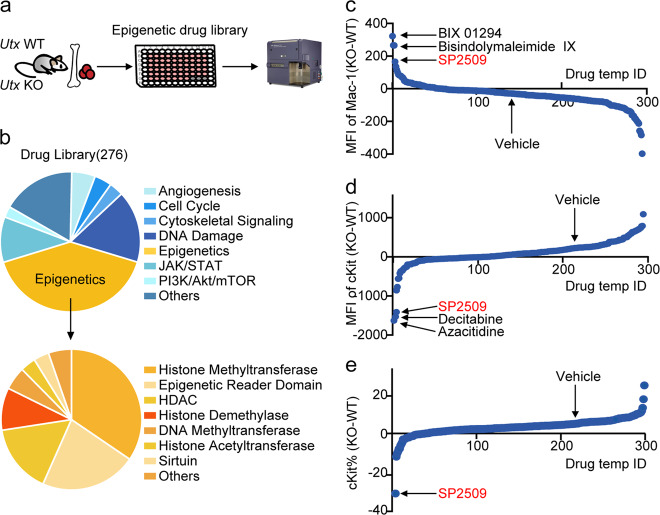
Epigenetic drug library screening for compounds specifically promoting the differentiation of Utx-null HSPCs. **a** Schematic showing the experimental design of epigenetic drug screening: HSPCs were isolated from young adult *Utx*^*f/f*^ (WT) or *Utx*^*f/f*^; *Mx-1-cre* (KO) C57BL/6 mice and then treated with compounds from an epigenetic drug library (Selleckchem, 276 compounds in total) individually. After 72-h treatment, cells were analyzed by flow cytometry with cKit and Mac-1 antibodies. **b** Summary of drug classification in the epigenetic drug library. The library consists of compounds directly targeting epigenetic enzymes and other related factors. **c**, **d** Relative mean fluorescence intensities (MFIs) of Mac-1 (**c**) and of cKit staining (**d**), measured by flow cytometry, in *Utx* WT and KO HSPCs treated with individual compounds from the epigenetic drug library or vehicle. **e** Relative proportions of cKit^+^ populations in *Utx* WT and KO HSPCs treated as in **c** and **d**

Given the functions of the tumor suppressor *Utx* in the differentiation of HSPCs through the modifications of histone 3, we decided to perform a high-throughput functional screening of an epigenetic drug library in wild-type (WT) and *Utx*-null HSPCs (Fig. 1a). We hypothesized that candidate small molecules could reverse the epigenetic abnormalities resulting from *Utx* loss and specifically release the differentiation block on *Utx*-deficient HSPCs. cKit^+^ HSPCs were isolated using MACS beads from wild-type (WT) or *Utx*^*f/f*^*;Mx1-cre* (KO) C57BL/6 mice^37,38^ 7 days after pIpC treatment. The epigenetic drug library has 276 compounds including a majority of currently available agonists and antagonists of epigenetic regulatory enzymes (Fig. 1b). The working concentrations of these drugs were 10 μM.

The outputs of the screen were stemness and differentiation status of HSPCs, measured by flow cytometry using cKit and Mac-1 staining, respectively. To identify compounds specifically enhancing the differentiation of *Utx* KO HSPCs while sparing WT HSPCs, we ranked the compounds by differences in the mean fluorescence intensity (MFI) of Mac-1 or cKit staining between *Utx* WT and KO HSPCs (Fig. 1c, d). Consistent with previous reports that *Utx* deficiency blocks HSPC differentiation, vehicle-treated *Utx* KO HSPCs expressed lower levels of Mac-1, a myeloid cell marker, than WT HSPCs, indicating impaired differentiation in KO cells. In contrast, *Utx* KO HSPCs expressed higher levels of cKit, a standard marker for HSPCs, than WT HSPCs.

The top three compounds that induced the most pronounced changes in Mac-1 expression between *Utx* KO and WT HSPCs were BIX01294, Bisindolylmaleimide IX, and SP2509 (Fig. 1c). BIX01294 is an inhibitor of histone methyltransferase G9a, and it therefore reduces H3K9m2 levels at an IC50 of 2.7 μM.^39^ Bisindolylmaleimide IX is a pan-PKC inhibitor, which can inhibit PKC-α, PKC-βI, PKC-βII, PKC-γ, and PKC-ε with low nanomolar IC_50_.^40^ SP2509 is a selective inhibitor of the histone demethylase LSD1 with an IC_50_ of 3 nM.^35,41^ On the other hand, the top three compounds that mostly reduced cKit expression in Utx KO HSPCs compared to that in WT HSPCs were azacytidine, decitabine, and SP2509 (Fig. 1d). Azacytidine and decitabine are already used in patients with various hematopoietic malignancies, and they induce differentiation of or directly kill leukemic blasts.^42,43^ The identification of azacytidine and decitabine validated our screening. However, we were more interested in the less studied SP2509, especially because it was the only compound identified to affect both Mac-1 and cKit expression. Actually, SP2509 was also the only candidate identified when the compounds were ranked by different proportions of cKit^+^ populations between *Utx* KO and WT HSPCs (Fig. 1e). Therefore, we focused on SP2509 in the subsequent studies.

### Validating SP2509 as a specific inducer of differentiation in *Utx*-deficient cells

**Fig. 2:**
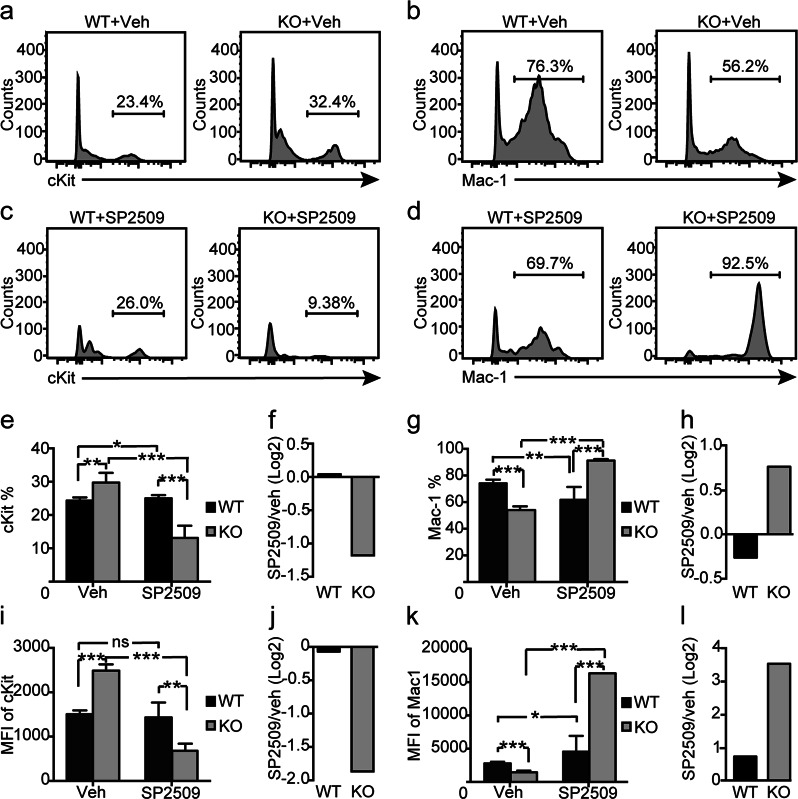
Validation of SP2509 in specifically inducing the differentiation of *Utx* KO HSPCs. **a**, **b** Representative flow plots showing cKit (**a**) and Mac-1 (**b**) staining in vehicle-treated *Utx* WT or KO HSPCs from three independent experiments. **c**, **d** Flow plots showing cKit (**c**) and Mac-1 (**d**) staining in SP2509-treated *Utx* WT and KO HSPCs. **e**–**h** Percentage of cKit^+^ (**e**) or Mac-1^+^ (**g**) populations in *Utx* WT and KO HSPCs treated with vehicle or SP2509. Corresponding fold changes of SP2509 vs vehicle-treated Utx WT or KO HSPCs are shown in **f** (cKit) and **h** (Mac-1). *n* = 3. **i**–**l** MFI of cKit (**i**) or Mac-1 (**k**) staining in *Utx* WT and KO HSPCs treated with vehicle or SP2509. Corresponding fold changes are shown in **j** (cKit) and **l** (Mac-1). *n* = 3. Error bars represent s.d. *p *<* 0.05; **p *<* 0.01; ***p *<* 0.001

To validate the function of SP2509 on *Utx* KO HSPCs, we performed another independent experiment similar to the library screening (Fig. 1a). First, in vehicle-treated group, *Utx* KO HSPCs remained more cKit^+^ cells than that in *Utx* WT HSPCs (32.4% vs 23.4%; Fig. 2a), while less Mac-1^+^ cells in *Utx* KO HSPCs than that in WT HSPCs (56.2% vs 76.3%; Fig. 2b), indicating that *Utx* loss impaired the differentiation of HSPCs. With SP2509 treatment, *Utx* WT HSPCs did not have much changes compared to vehicle-treated group. Twenty-six percent of SP2509-treated *Utx* WT HSPCs remained cKit^+^, whereas 69.7% became Mac-1^+^ (Fig. 2c, d). In striking contrast, only 9.38% of *Utx* KO HSPCs remained cKit^+^ after SP2509 treatment, which was approximately 2.5-fold lower than that in vehicle-treated KO HSPCs and significantly less than that in WT HSPCs (Fig. 2e, f). Conversely, 90% of *Utx* KO HSPCs became Mac-1^+^ with SP2509 treatment, which was approximately one-fold higher than that in vehicle-treated *Utx* KO HSPCs and significantly more than that in WT HSPCs (Fig. 2g, h). Consistently, the MFI of cKit staining in *Utx* KO HSPCs decreased from 2489 for vehicle treatment to 681 for SP2509 treatment, a 2.65-fold reduction, while there was no significant difference between vehicle- and SP2509-treated WT HSPCs (Fig. 2i, j). The MFI of Mac-1 staining in *Utx* KO HSPCs increased from 1450 for vehicle treatment to 16,326 for SP2509 treatment, a 10.2-fold increase, which was dramatically more than the difference between vehicle- and SP2509-treated *Utx* WT HSPCs (Fig. 2k, l). Therefore, SP2509 was validated as a specific differentiation inducer for *Utx* KO HSPCs while sparing WT HSPCs.

### SP2509 treatment reversed the global effect of *Utx* deficiency on the transcriptome of HSPCs

**Fig. 3:**
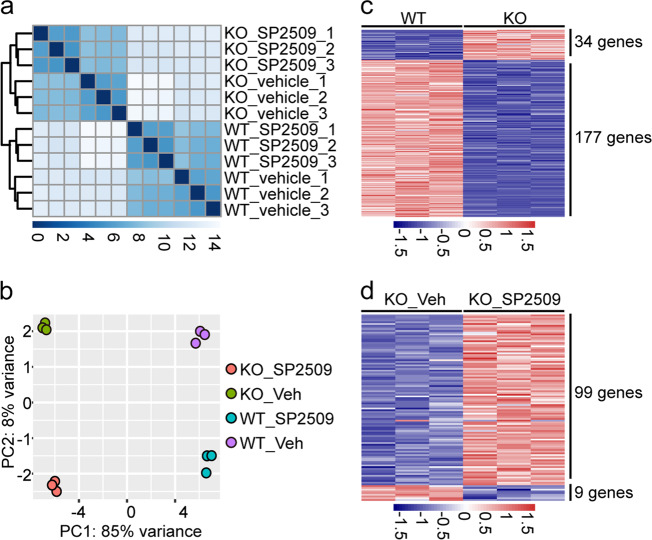
SP2509 treatment reversed the global effect of *Utx* deficiency on the transcriptome of HSPCs. **a**, **b** Unsupervised clustering (**a**) and principal component analysis (PCA) (**b**) of RNA-seq data from *Utx* WT and KO HSPCs treated with vehicle or SP2509 for 12 h. **c** Heatmap of differentially expressed genes in *Utx* WT or KO HSPCs measured by RNA-seq. **d** Heatmap of differentially expressed genes in *Utx* KO HSPCs treated with vehicle or SP2509 for 12 h

To understand the molecular mechanisms underlying the SP2509-induced differentiation of *Utx* KO HSPCs, we used RNA sequencing (RNA-seq) to analyze the transcriptomes of *Utx* WT and KO HSPCs treated with vehicle or SP2509. Cells were harvested after 12-h treatment to capture the direct effects of SP2509 treatment on gene expression. In each group, there were three independent treated samples. Unsupervised grouping analysis showed that all of the three samples were grouped together in each group (Fig. 3a), indicating a high quality of the RNA-seq data. Principal component analysis (PCA) also revealed tight grouping of the three replicates in each group. The location of *Utx* WT and KO HSPCs treated with vehicle or SP2509 on the PCA plot clearly demonstrated the differences between *Utx* WT and KO HSPCs and those between vehicle- and SP2509-treated HSPCs (Fig. 3b).

Interestingly, compared to the differentially expressed genes in WT HSPCs, 177 genes were significantly downregulated in *Utx* KO HSPCs (*p* < 0.05, log2[KO/WT] < −1), while only 34 genes were significantly upregulated (Fig. 3c). The overwhelming repression of gene expression is consistent with the histone modification functions of Utx and its associated COMPASS-like complex. Similar results were also observed in Mll3 (the core component of COMPASS-like complex)-deficient HSPCs.^8^

In striking contrast, 99 genes in SP2509-treated *Utx* KO HSPCs were significantly upregulated (*p* < 0.05, log2[KO/WT] < −1) compared to those in vehicle-treated *Utx* KO HSPCs, while only nine genes were significantly downregulated (Fig. 3d). Notably, the overwhelming upregulation of gene expression by SP2509 was directly contradictory to the effect of *Utx* loss in HSPCs. Accordingly, the effects of SP2509 treatment on the global transcriptome was specific to *Utx* KO HSPCs, and the up- and downregulated genes in SP2509-treated vs vehicle-treated *Utx* WT HSPCs were largely balanced.

### SP2509 enhanced the expression of differentiation-related genes and tumor suppressors repressed in *Utx* KO HSPCs

**Fig. 4:**
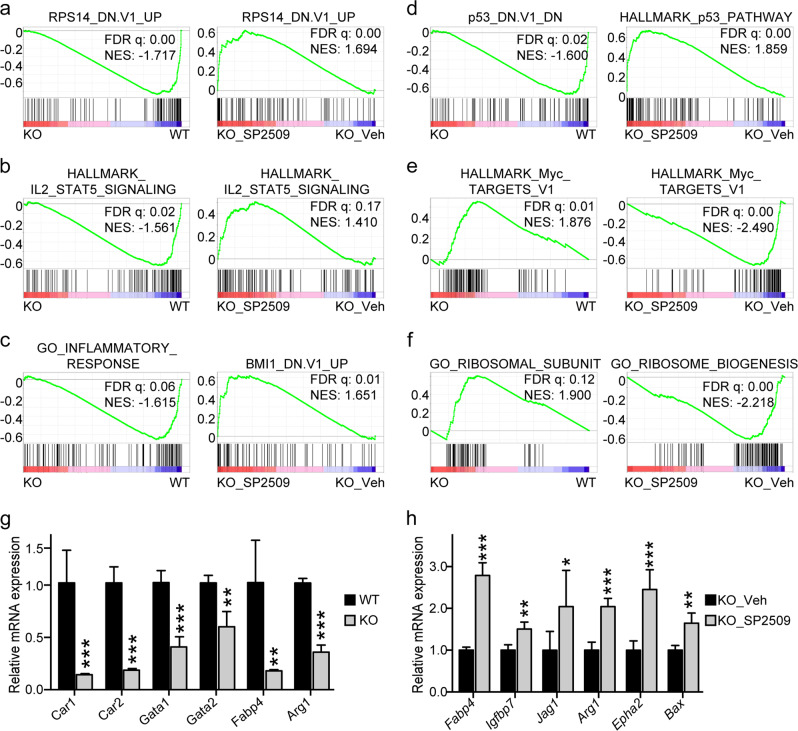
*Utx* deficiency repressed the expression of differentiation-related genes and tumor suppressors, while SP2509 treatment reversed this inhibition. **a**–**f** Gene set enrichment analysis (GSEA) of genes in the RPS14_DN. V1_UP gene set (**a**), the Hallmark_IL2_STAT5_signaling gene set (**b**), the GO_Inflammatory_Response and the BMI1_DN. V1_UP gene set (**c**), the p53_DN. V1_DN, and the Hallmark_p53_pathway gene set (**d**), the Hallmark_Myc_Targets_V1 gene set (**e**), the GO_Ribosomal_Subunit and the GO_Ribosome_Biogenesis gene set (**f**) in *Utx* KO HSPCs compared to that in *Utx* WT HSPCs, and *Utx* KO HSPCs treated with SP2509 compared to that in cells treated with vehicle (Veh). **g** Relative mRNA expression level of *Car1, Car2, Gata1, Gata2, Fabp4*, and *Arg1* in *Utx* WT or KO HSPCs, measured by qPCR. *n* = 3. **h** Relative mRNA expression of *Fabp4, Igfbp7, Jag1, Arg1, Epha2*, and *Bax* in *Utx* KO HSPCs treated with vehicle (Veh) or SP2509. *n* = 3. Error bars represent s.d. *p *<* 0.05; **p *<* 0.01; ***p *<* 0.001

To understand the functions of SP2509-regulated genes, we performed gene set enrichment analysis (GSEA). Consistent with the differentiation block due to *Utx* deficiency^44^ (Fig. 2a–d), hematopoietic cell lineage-related pathways and multiple immune-related pathways were the most enriched pathways among the downregulated genes in *Utx* KO HSPCs. Transcriptional signatures produced by *Utx* KO (compared to *Utx* WT) were negatively correlated with the RPS14 pathway^45^-associated gene signature (normalized enrichment score [NES] = −1.717, false discovery rate [FDR] = 0.004). Nevertheless, the transcriptional signatures in response to SP2509 treatment were positively correlation with the RPS14 pathway ([NES] = 1.694, [FDR] = 0.006) (Fig. 4a). Similarly, the hallmark IL2/STAT5 pathway-related gene set was negatively enriched in *Utx* KO HSPCs ([NES] = −1.561, [FDR] = 0.017). In turn, this gene set was positively enriched in *Utx* KO HSPCs treated with SP2905 ([NES] = 1.410, [FDR] = 0.170) (Fig. 4b). Further, the transcriptional signature of *Utx* KO HSPCs was negatively enriched in genes involved in the inflammatory response pathway (NES = −1.615, FDR *q* = 0.013), while the Bmi1_DN.V1_UP^46^ gene set, related to stemness, was enriched in SP2590-treated *Utx* KO HSPCs (NES = 1.651, FDR *q* = 0.010). Hence, Utx deficiency inhibited the expression of differentiation-related genes, which was reversed by SP2509 treatment.

Interestingly, the transcriptional signature produced by *Utx* KO was negatively correlated with the tumor suppressor p53 pathway-related gene set ([NES] = −1.600, [FDR] = 0.017) and positively correlated with the Myc pathway-related gene set ([NES] = 1.876, [FDR] = 0.006), which might explain the functions of Utx in numerous human cancers.^27,30,31,44,47^ In contrast, the transcriptome in SP2509-treated KO HSPCs was significantly positively enriched in p53 pathway-related genes ([NES] = 1.859, [FDR] = 0.000) and negatively correlated with Myc pathway-related genes ([NES] = −2.490, [FDR] = 0.000) (Fig. 4e, f). Additionally, the ribosome biogenesis-related gene set was positively enriched in *Utx* KO HSPCs ([NES] = 1.90, [FDR] = 0.12). Conversely, this gene set was negatively enriched in SP2590-treated *Utx* KO HSPCs ([NES] = 1.90, [FDR] = −2.218) (Fig. 4f). Thus, SP2509 treatment rescued the expression of tumor suppressors and oncogenes perturbed by *Utx* deficiency in HSPCs.

Quantitative PCR analysis of selected differentiation-related genes, such as *Car1*, *Car2*, *Gata1,* and *Gata2*, from GSEA in *Utx* KO HSPCs confirmed significant decreased expression compared to that in *Utx* WT HSPCs (*p* < 0.01) (Fig. 4g). Accordingly, SP2509-treated *Utx* KO HSPCs displayed significantly increased expression of selected differentiation-related genes and tumor suppressors including *Bax* compared to vehicle-treated *Utx* KO HSPCs (*p* < 0.05) (Fig. 4h).

### SP2509 treatment reversed the H3K4 methylation-mediated repression of differentiation-related genes and tumor suppressors in *Utx* KO HSPCs by inhibiting LSD1

**Fig. 5:**
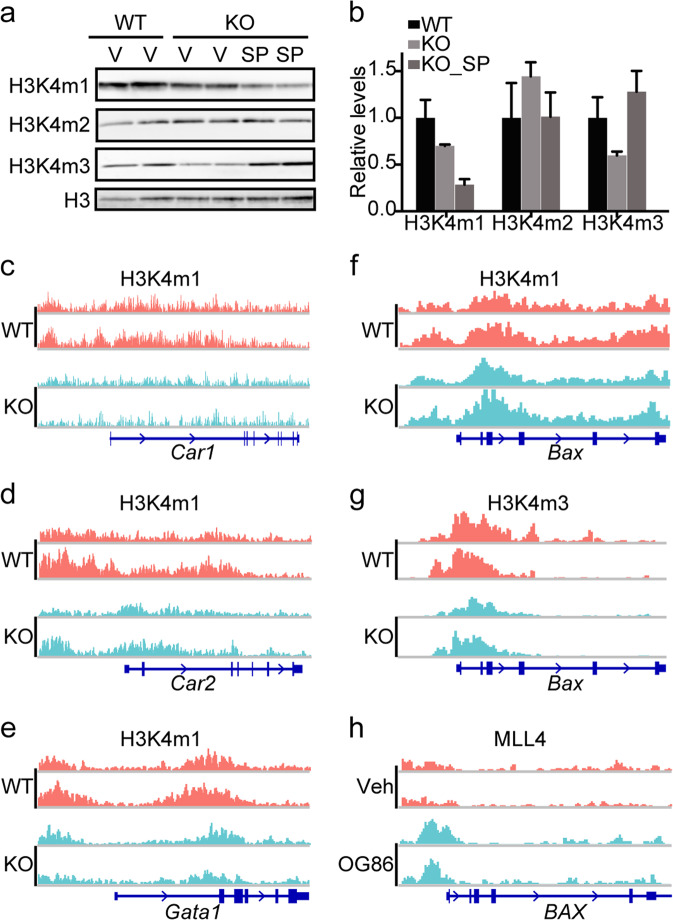
SP2509 treatment reversed *Utx* deficiency inducing the histone H3K4 methylation change on globe and specific differentiation-related or tumor suppressor genes in HSPCs. **a** Western blotting of H3K4m1, H3K4m2, and H3K4m3 in cell lysates from *Utx* WT or KO HSPCs treated with vehicle (V) or SP2509 (SP) for 12 h. **b** Quantification of H3K4m1, H3K4m2, and H3K4m3 levels in **a**. **c**–**f** H3K4m1 peaks on *Car1*(**c**), *Car2* (**d**), *Gata1*(**e**), and *Bax* (**f**) in *Utx* WT or KO HSPCs measured by ChIP-seq. Data analyzed from Gozdecka et al.^44^ **g** H3K4m3 peaks on *Bax* in *Utx* WT and KO ES cells measured by ChIP-seq. Data analyzed from Dhar et al.^49^ **h** MLL4 peaks on *BAX* in vehicle or OG86-treated leukemic cells measured by ChIP-seq. Data analyzed from Maiques-Diaz et al.^50^

Given the H3K4 methylation activity of Utx and its associated COMPASS-like complex and the H3K4 demethylation activity of LSD1, the putative target of SP2509,^48^ we further studied the mechanisms of SP2509-induced differentiation in *Utx* KO HSPCs. As shown in western blot (Fig. 5a, b), the total H3K4m3 level was reduced in *Utx* KO HSPCs compared to that in *Utx* WT HSPCs. Although Utx itself does not have a direct H3K4-modifying activity, it associates with Mll3/4 in COMPASS-like complex, which are H3K4 mono- and dimethyltransferase. Actually, we did observe a significant reduction in H3K4m1 levels, but the levels of H3K4m2 were similar between *Utx* KO and WT HSPCs. Consistently, differentiation-related genes, such as *Car1*, *Car2* and *Gata1*, and the tumor suppressor *Bax*, displayed reduced H3K4m1 levels in *Utx* KO HSPCs compared to those in WT HSPCs^44^ (Fig. 5c–f), which led to the decreased expression levels of these genes (Fig. 4h). Our results suggested that Utx loss might impair the H3K4 methyltransferase activities of Mll3/4.

In contrast, LSD1, the target of SP2509, is an H3K4 and H3K9 demethylase. Indeed, LSD1 inhibition by SP2509 increased H3K4m3 levels in *Utx* KO HSPCs (Fig. 5a, b). The total levels of H3K4m2 were not significantly changed in *Utx* KO HSPCs treated with SP2509, but the level of H3K4m1 was further reduced, suggesting that a majority of these loci might have been further methylated to H3K4m3. The level of H3K4m3 in *Bax* gene was reduced in *Utx* KO ES cells compared to that in *Utx* WT cells^49^ (Fig. 5g). Interestingly, treatment with OG86, another LSD1 inhibitor, increased the binding of MLL4 to the promoter of *BAX* in leukemic cells^50^ (Fig. 5h), suggesting that LSD1 and the COMPASS-like complex might directly compete for binding on the promoters of differentiation-related genes and tumor suppressors. Thus, LSD1 inhibition reversed the reduction in H3K4 methylation in *Utx*-deficient cells and increased the expression of differentiation-related genes and tumor suppressors.

### SP2509 treatment promoted the differentiation of *Utx*-deficient AML cells in vitro and in vivo and extended the survival of leukemic mice

**Fig. 6:**
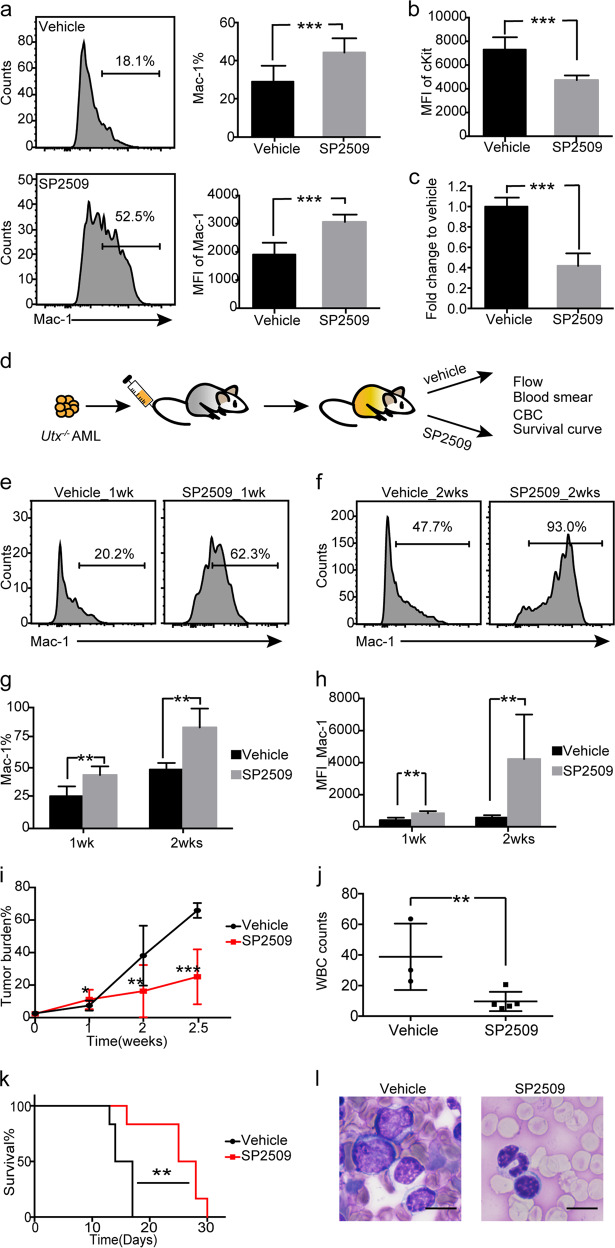
SP2509 inhibited *Utx*-mutated AML by promoting differentiation in vitro and in vivo. **a** Flow cytometry measuring protein expression in *Utx*^−*/−*^; *shp53-mCherry;shNf1-GFP* AML cells treated with vehicle or SP2509. Left, representative flow plots of AML cells showing the expression of Mac-1. Right, percentage of Mac-1^+^ populations (top), and the MFI of Mac-1 staining (bottom). **b** MFI of cKit staining in AML cells treated with vehicle or SP2509. **c** Relative cell number of AML cells treated with vehicle or SP2509. **d** Schematic showing *Utx*^*−/−*^; *shp53-mCherry;shNf1-GFP* mice with AML treated with vehicle or SP2509. C57BL/6 mice were sublethally irradiated and then transplanted with *Utx*^*−/−*^; *shp53-mCherry; shNf1-GFP* AML cells. Once AML was established, these mice were treated with vehicle or 25 mg/kg SP2509 twice per week via i.p. injection. The mice were monitored by flow cytometry, CBC, and blood smear. **e**, **f** Representative flow plots showing the expression of Mac-1 in peripheral blood AML cells in vehicle- or SP2509-treated mice after 1 (**e**) or 2 (**f**) weeks. **g**, **h** Percentage of Mac-1^+^ (**g**) and MFI of Mac-1 staining (**h**) of AML cells in the peripheral blood of vehicle- or SP2509-treated mice. **i** Tumor burden in the peripheral blood of vehicle- or SP2509-treated mice, measured by the percentage of GFP^+^mCherry^+^ populations. **j** Whole blood cell counts in vehicle- or SP2509-treated mice after 2.5 weeks. **k** Kaplan–Meier survival curves of mice with AML treated with vehicle or SP2509. *n* = 6, **p < 0.01. **l** Blood smear showing leukemic cells in the peripheral blood of vehicle- or SP2509-treated mice

We further tested whether SP2509 would have similar inhibitory effect on *Utx*-deficient malignant cells as it did on *Utx* KO HSPCs. *Utx*^*−/−*^; *shp53-mCherry;shNf1-GFP* AML cells (from unpublished independent work) were treated with or without 10 μM SP2509 for 3 days. By flow cytometry, we found that SP2509 treatment dramatically increased the expression of the differentiation marker Mac-1 in leukemia cells (Fig. 6a). The percentage of Mac-1^+^ population and the MFI of Mac-1 staining in SP2509-treated *Utx*-null AML cells were approximately two-fold higher than those in vehicle-treated cells. Accordingly, the expression of the stemness marker cKit was reduced by SP2509 (Fig. 6b). Consistent with the release of differentiation block, SP2509 significantly repressed the growth of *Utx*-null AML cells (Fig. 6c). Thus, similar to its function in premalignant *Utx*-deficient HSPCs, SP2509 inhibited *Utx*-null AML cells in vitro by promoting their differentiation. These data validated our screening strategy to target *Utx* deficiency in malignant cells.

Then, the potential therapeutic effect of SP2509 on *Utx*-deficient AML was tested in vivo. *Utx*^−*/−*^; *shp53-mCherry;shNf1-GFP* AML cells were transplanted into sublethally irradiated congenic recipient mice. Seven days after transplantation, all of the recipient mice had similar proportions of GFP^+^-mCherry^+^ AML cells in their peripheral blood measured by flow cytometry. These leukemic mice were randomly assigned into two groups: one was treated with 25 mg/kg SP2509 twice per week by intraperitoneal injection, while the other one was treated with vehicle. These mice were monitored for disease progression by complete blood cell (CBC) count, blood smear staining, and flow cytometry (Fig. 6d). One week after treatment, we observed approximately 50% Mac-1^+^ AML cells in the peripheral blood of SP2509-treated mice compared to 25% in the peripheral blood of vehicle-treated mice (Fig. 6e). This differentiation-promoting effect of SP2509 was more obvious after a 2-week treatment. The majority of AML cells were Mac-1^+^ in SP2509-treated mice, and the MFI of Mac-1 staining in SP2509-treated leukemic cells was approximately five-fold higher than that in vehicle-treated cells (Fig. 6f–h). We did not observe significant weight loss in mice treated with SP2509, suggesting that SP2509 was well tolerated by the mice (data not shown). These data demonstrated that SP2509 promoted the differentiation of *Utx*-mutated tumor cells in vivo.

More importantly, SP2509 prevented the progression of AML in vivo. The tumor burden of recipient mice was measured using the percentage of GFP^+^-mCherry^+^ cells in peripheral blood. *Utx*-mutated AML was very aggressive, and leukemic cells progressively proliferated in vehicle-treated mice. The tumor burden in this group increased from 2% to 70% in 2.5 weeks. In contrast, tumor burden increased much slower in SP2509-treated mice, as only 20% AML cells detected in the peripheral blood after 2.5 weeks (Fig. 6i). Interestingly, more leukemic cells in SP2509-treated mouse peripheral blood were observed than that in vehicle-treated mice 1 week after treatment. This caveat might be because of early proliferation associated with differentiation, which we observed within other AML models, such as IDH2-mutated AML.^6^ After 2.5-week treatment, whole blood cell count in vehicle-treated mice were 25–65k/μL, but those in SP2509-treated mice were largely normal (Fig. 6j). Furthmore, all of the vehicle-treated mice died within 17 days after treatment, while five out of six SP2509-treated mice survived over 3 weeks (Fig. 6k). The surviving mice had few blast cells in their peripheral blood (Fig. 6l). Thus, SP2509 exerted marked therapeutic effects on *Utx*-mutated tumors in vivo.

**Fig. 7:**
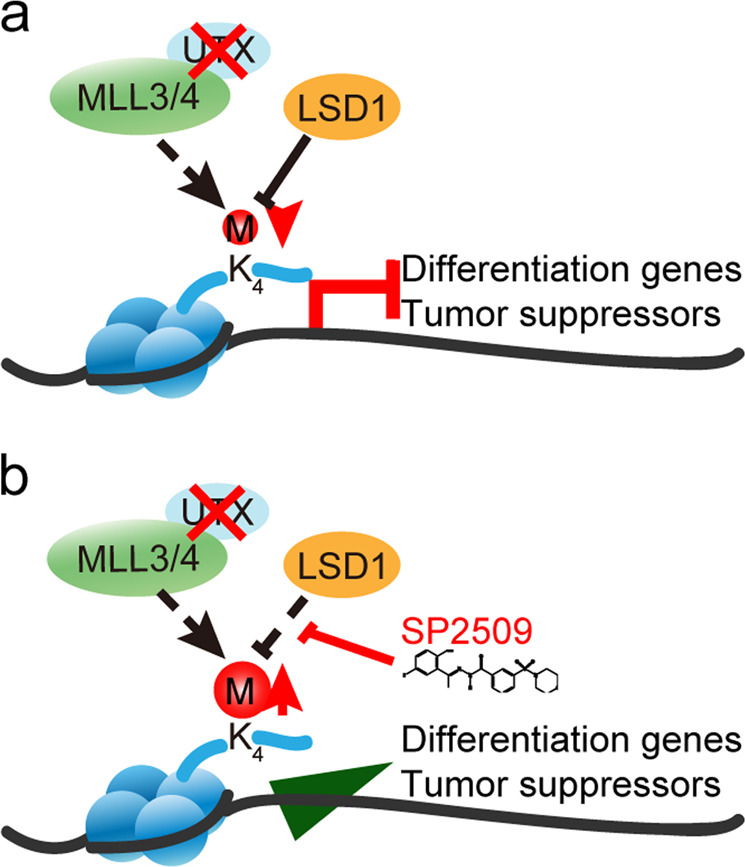
A working model of the molecular mechanism by which SP2509 induces the differentiation of Utx-deficient cells. **a** Schematic showing that the H3K4 methylation activity of MLL3/4 in the COMPASS-like complex is impaired by Utx deficiency, which leads to a reduced expression of differentiation-related genes and tumor suppressors. **b** Schematic showing that in Utx-deficient cells, the small molecule SP2509 inhibits the demethylation activity of LSD1 on H3K4 and therefore rescues the expression of differentiation-related genes and tumor suppressors

In summary, *Utx* loss reduces H3K4 methylation, presumably through the COMPASS-like complex, and therefore represses the expression of differentiation-related genes and tumor suppressors, which leads to a differentiation block in HSPCs eventually giving rise to tumorigenesis (Fig. 7a). LSD1 competes with MLL3/4 on the promoters of these genes. Thus, the inhibition of LSD1 by SP2509 reverses the reduction in H3K4 methylation in *Utx*-deficient cells, which gives rise to an increased expression of differentiation-related genes and tumor suppressors and specifically promotes the differentiation of *Utx*-mutated cells (Fig. 7b).

## Discussion

Accumulating evidence suggests that epigenetic abnormalities are common and critical for human cancers, especially hematopoietic malignancies. Many cancer-associated epigenetic alterations promote tumorigenesis by impairing the differentiation of tissue stem and/or progenitor cells. Thus, differentiation therapy would be especially effective for cancers involving epigenetic dysregulation. Since any given epigenetic modification, including histone methylation, can regulate the expression of a number of targets, the direct reversal of epigenetic abnormalities due to a driver epigenetic modifier would be more efficient than targeting individual targets. The balance between epigenetic writers and erasers provides opportunities to identify therapeutic targets specific for cancers with given epigenetic abnormalities.

UTX, an H3K27 demethylase and a key component of the COMPASS-like complex, is a putative tumor suppressor in multiple human cancers. Our study, as well as previous reports, showed that *UTX* deficiency impairs the differentiation of HSPCs. In this study, we screened an epigenetic drug library using *Utx* KO and WT HSPCs. We found that SP2509, an inhibitor of the H3K4 and H3K9 demethylase LSD1, specifically promoted the differentiation of *Utx*-null HSPCs while sparing *Utx* WT HSPCs. Based on the effect of SP2509 on *Utx*-deficient premalignant cells, we further tested it in *Utx*-mutated tumor cells in vitro and in vivo. Notably, SP2509 had similar differentiation-promoting effect on AML cells, and, more importantly, it inhibited leukemia progression in vivo and significantly extended the lifespan of mice with AML. Mechanistically, *Utx* mutations repress the expression of differentiation-related genes and tumor suppressors by impairing H3K4 methylation on these genes, while SP2509 inhibits LSD1 and reverses the impairments in H3K4 methylation in *Utx*-mutated cells. Thus, we identified a highly specific inhibitor for abnormal cells with deficiencies in *Utx* and/or the COMPASS-like complex. Interestingly, SP2509 and some other LSD1 inhibitors have been suggested to be effective in some leukemias and other human cancers.^41,51^ SP2577, the clinical formulation of SP2509, is in a phase I clinical trial for patients with relapsed or refractory Ewing sarcoma. Further validation of SP2509 and other inhibitors with similar functions in human cancer cells with UTX mutations or COMPASS-like complex dysfunctions would pave the way for their potential applications for the treatment of patients with such epigenetic abnormalities.

## Methods

### Mice

*Utx*^*f/f*^ mice (purchased from The Jackson Labratory, 024177) were crossed with Mx1-Cre mice (purchased from Jackson Lab, 005673). The expression of Cre was induced by the intraperitoneal injection of pIpC (Sigma P1530, 10 mg/kg) into 5- to 6-week-old mice every other day for a total of 10 days.

### Isolation of HSPCs and cell culture

WBM cells were freshly isolated from *Utx*^*f/f*^ and *Utx*^*f/f*^; *Mx1-Cre* mice, and then HSPCs were sorted with mouse CD117 magnetic microbeads (Miltenyi Biotec, 130-091-224). Primary mouse cells were cultured in BCM medium (50% IMDM + 50% DMEM + 10% FBS + 5% PS + 0.34% BME) with 10% Stem Cell Medium (SCM) including 10% FBS, IL3 (10 ng/mL; 1:1000), IL6 (10 ng/mL; 1:500), and SCF (50 ng/mL; 1:1000).

### Drug screening

The epigenetics compound library (including 276 compounds) used for drug screening was purchased from Selleckchem (Catalog# L1900). The compounds were generally provided in DMSO at 10 μM concentrations. After HSPCs were isolated from bone marrow and cultured in vitro for 24 h, they were seeded at equal numbers in five low-attachment 96-well plates (the primary number of cells was 40,000 in 100 μL medium per well; in one 96-well plate, 60 wells were filled with cells, while other surrounding wells were filled with PBS). Then, 276 kinds of compounds at a final concentration of 10 μM were used separately (marked in pink), and DMSO was used to treat control cells (marked in gray). After 3 days of treatment, cell features were determined by flow cytometry.

### Flow cytometry

Cells were stained with antibodies against Mac-1 (Clone: M1/70, BD101224) and cKit (Clone: 2B8, BD105812) after 3 days of treatment. All flow cytometry analyses were performed with an LSR Fortessa instrument (BD), and the data were analyzed using FlowJo software.

### RNA-sequencing analysis

RNA-seq libraries were prepared using NEBNext® Ultra™ RNA Library Prep Kit for Illumina® and were sequenced with an Illumina HiSeq™ X sequencing machine with 150-bp paired-end reads. The RNA-seq reads were aligned to a reference genome (GRCm38) by STAR_2.6.0a.^52^ Transcript abundance was normalized and measured in fragments per kb of exon per million fragments mapped (FPKM). Differential gene expression was analyzed by DESeq2.^53^ Genes with absolute fold changes greater than 0.5 and FDR ≤ 0.05 were considered differentially expressed genes. Heatmaps of differentially expressed genes were constructed and normalized by *Z* scores. Samples distances were calculated by PCA and the Euclidean distance. GSEA^54,55^ was employed to determine statistically significant similarities and differences between two given clusters by identifying a priori-defined gene sets.

### ChIP-seq data collection and visualization

Data were downloaded from the GEO database under accession codes GSE63222,^50^ GSE101307,^44^ and GSE76692.^49^ In addition, data were visualized by Integrative Genomics Viewer^56^ (IGV).

### Western blotting

1M cells were harvested and lysed in SDS buffer (50 mM Tris-HCl (pH 6.8), 2% (w/v) SDS, 150 mM NaCl, 1% NP-40, 40 mM DTT) followed by sonication with an Ultrasonic Cell Disruptor. Then, lysate proteins were separated by 15% SDS–PAGE gels and then transferred to PVDF membranes. Western blots were performed using antibodies against H3K4m1 (Abcam, ab8895), H3K4m2 (Abcam, ab7766), H3K4m3 (Abcam, ab8580), or total histone 3 (H3, HuaBio, EM30605).

### qPCR assay

Expression levels of genes (one set of the genes was selected from downregulated genes in the microarray data comparing the *Utx* KO group to the WT group, and the other set of genes was selected from upregulated genes in the microarray data comparing SP2509 treatment to vehicle treatment in the *Utx* KO group) were quantified by using SYBR Green Master Mix and the Quant Studio 3 Real-Time PCR System (Thermo Fisher Scientific). qPCR primer sequences are listed in Table 1.

**Table 1:** qPCR primer sequences

Gene name	Primer sequence
Car1_F	CAAGCCTGCAGAAAGTACTTG
Car1_R	CCAAAGTAGGTCCAGTAATCCAG
Car2_F	GGCCTTCAGAAAGTCCTTGA
Car2_R	GGGAGCAAGGATCGAAGTTAG
Gata1_F	AAGATGAATGGTCAGAACCGG
Gata1_R	GTTTGACAGTTAGTGCATTGGG
Gata2_F	GGGCTCTACCACAAGATGAATG
Gata2_R	TCGTCTGACAATTTGCACAAC
Fabp4_F	GAAGCTTGTCTCCAGTGAAAAC
Fabp4_R	GACCAAATCCCCATTTACGC
Igfbp7_F	CCTGTCCTCATCTGGAACAAG
Igfbp7_R	CCCGTTACTTCATGCTTTTCTG
Epha2_F	TGCCAGCGTCAGTATTAACC
Epha2_R	GTAGGTGACTTCGTACTTCCAC
Bax_F	CTGACATGTTTGCTGATGGC
Bax_R	GAAGTCCAGTGTCCAGCC
Jag1_F	TGTAAACTTCCAGGTGACTGC
Jag1_R	CAGTTGGTCTCACAGAGGC
Arg1_F	GTAGAGAAAGGCCCTGCAG
Arg1_R	GAAAGGAGCTGTCATTAGGGAC

### Leukemia modeling and in vivo treatment

All animal studies were approved by the Institutional Animal Care and Use Committees of Sichuan University. Approximately 2 × 10^6^ *Utx*^*−/−*^*; shp53-mCherry;shNf1-GFP* AML cells were transplanted by tail-vein injections into sublethally irradiated (4.5 Gy) 8-week-old female C57BL/6 mice. Drug treatments were initiated on day 7 after the cells were transplanted, and six mice were treated in each group: vehicle vs 25 mg/kg SP2509. SP2509 in solvent buffer (20% PEG-40, 20% dimethyl sulfoxide, 60% sterile water) or vehicle was intraperitoneally administered twice per week (Tuesday and Thursday) for 3 weeks as previously administrated.^41^ The leukemia progression in recipient mice was monitored by CBC, flow cytometry, and blood smear once per week.
